# What Is New in *Helicobacter pylori* Diagnosis. An Overview

**DOI:** 10.3390/jcm10102091

**Published:** 2021-05-13

**Authors:** Maria Pina Dore, Giovanni Mario Pes

**Affiliations:** 1 Dipartimento di Scienze Mediche, Chirurgiche e Sperimentali, University of Sassari, 07100 Sassari, Italy; gmpes@uniss.it; 2 Baylor College of Medicine, One Baylor Plaza, Houston, TX 77030, USA

**Keywords:** *Helicobacter pylori*, testing, antibiotic resistance, molecular techniques, artificial intelligence

## Abstract

*Helicobacter pylori* infection remains one of the most prevalent infections worldwide, especially in low-resource countries, and the major risk factor for peptic ulcer and gastric cancer. The “test-and-treat” strategy is recommended by several guidelines and consensus. The choice of testing method is based on patient age, presence of alarm signs and/or symptoms, use of non-steroidal anti-inflammatory drugs, as well as local availability, test reliability, and cost. Culture is the gold standard to detect *H. pylori* and, possibly, to perform susceptibility testing, however, it requires upper endoscopy and dedicated labs. Recent advances in molecular biology have provided new strategies in detecting infection and antimicrobial resistance without invasive tests. In this review we attempt to offer a comprehensive panorama on the new diagnostic tools and their potential use in clinical settings, in order to accomplish specific recommendations.

## 1. Introduction

*Helicobacter pylori* infection can be essentially detected by invasive and non-invasive tests. The choice of technique relies upon the patient’s needs. Presence of alarm symptoms, use of non-steroidal anti-inflammatory drugs (NSAIDs), advanced age (>45–50 years or >60 years) [1,2,3,4], history of premalignant conditions, or surveillance for a previous malignant disease dictates an upper endoscopy evaluation. The indication for esophago-gastric-duodenoscopy allows physicians to directly observe the mucosa, to collect biopsy samples for histology examination, urease test, bacterial culture and, eventually, molecular assay. In the absence of endoscopy recommendation, non-invasive tests, such as urea breath testing or stool antigen assay, are appropriate to confirm an active infection. Serology may be used in specific settings to assist the physician in the diagnosis of bacterial infection [5]. However, the diagnostic strategy cannot prescind from the local availability, costs of the test, and the patient’s preferences.

## 2. Invasive Tests

### 2.1. Endoscopy

Since the first isolation of *H. pylori*, several studies have attempted to evaluate the accuracy of standard white light endoscopy (WLE) to identify the infection, based on specific gastric mucosa features. For example, the presence of antral nodularity, observed during endoscopy, was associated with a sensitivity ranging from 39.8% [6] to 96.4% [7] and a specificity ranging from 83.6% [6] to 100% [8]. Additional reports identified the erythema, erosions, thickened folds or absence of rugae, mosaic appearance, with or without hyperemia, and visible submucosal vessels in the gastric mucosa as the hallmarks of *H. pylori* infection [9,10,11,12], or gastric black spots associated with *H. pylori* eradication [13]. Moreover, a study performed in Japan to evaluate the accuracy of standard endoscopy found that nodularity (89%) and mucosal swelling (77%) were associated with bacterial infection and mild atrophy [14]. However, the low interobserver agreement may be a limitation to translate gastric mucosal features into a diagnosis of specific gastritis, with or without *H. pylori* infection.

The results obtained with the narrow band imaging (NBI), which uses blue light from a laser source (415 nm) to highlight the vascular architecture of the gastric mucosa, seem to be more promising. Tongtawee et al. were able to predict *H. pylori* infection based on distinct patterns of gastric mucosa, observed by conventional NBI [15]. In addition, the magnifying NBI technique showed a sensitivity and specificity greater than 95% in detecting intestinal metaplasia [16], especially when a light blue crest or white opaque substance were present [17], and proved to be significantly superior (*p* < 0.0001) to serology (pepsinogen I/II ratio) [18]. Moreover, a high degree of concordance was observed between magnifying NBI and the operative link for gastritis and for gastric intestinal metaplasia assessment [19,20]. Interestingly, by this technique, specific morphological patterns, including reddish depressed lesions, were frequently observed in association with *H. pylori* eradication [16,21]. The magnifying endoscopy with NBI also proved to be superior to WLE and chromoendoscopy in the diagnosis of early gastric cancer, after *H. pylori* eradication [22]. However, Horiguchi et al. reported that the confocal laser endomicroscopy was more accurate than NBI for grading gastric premalignant lesions [23].

In a retrospective study conducted to detect gastric atrophy, by using blue laser imaging (BLI), the presence of a spotty pattern was associated with an active *H. pylori* infection, the cracked pattern with *H. pylori* eradication, and the mottled pattern with intestinal metaplasia [24]. However, the linked color imaging (LCI) results were superior to BLI in the recognition of early gastric cancer and *H. pylori*-negative gastritis [25]. The LCI, by improving the visualization of mucosal microstructure via contrast enhancement, was able to detect an active, or past *H. pylori* infection and associated lesions with high accuracy, although the accuracy was different for gastritis, metaplasia or atrophy [26,27,28]. In a comparison study between LCI and magnifying BLI-bright, the authors found the former technique highly accurate for *H. pylori* infection, and the latter for atrophy and intestinal metaplasia [29].

Endocytoscopy (EC), an ultra-high magnification endoscopy, is able to provide a histologic assessment in vivo. Sato et al. observed that EC patterns, such as normal pit-dominant type, or the normal papilla-dominant type, visualized in the corpus and antrum, were hallmarks of normal mucosa and of the absence of *H. pylori* infection [30]. In recent years, an in vivo method was also developed, based on in situ hybridization fluorescence, enabling the diagnosis of active infection during endoscopy [31].

All recent developments of high-definition endoscopy for the diagnosis of *H. pylori* infection and detection of pre-malignant and malignant gastric lesions, allowing real-time decision-making, prompted the revision of the Kyoto endoscopic classification [32].

**Figure 1:**
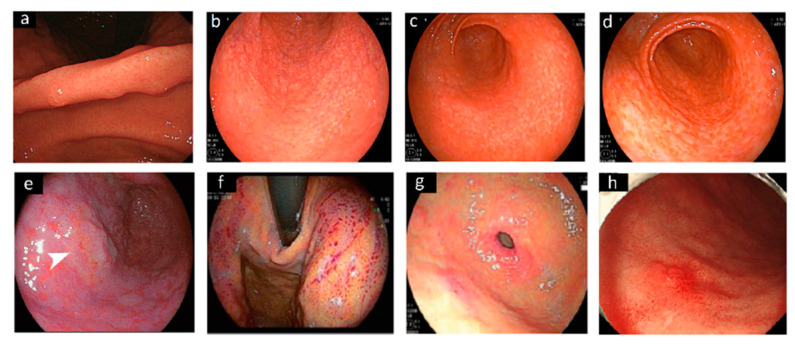
Images of different endoscopic patterns related to *H. pylori* infection. Specific features of the gastric angulus (**a**) in a 30-year-old man *H. pylori*-positive [14]. Gastric antrum showing (**b**) a “spotty pattern”, (**c**) a “cracked pattern” and (**d**) a “mottled pattern” observed by white light endoscopy [24]; (**e**) orange lesion (arrow), suggestive of early gastric cancer in the antrum, surrounded by spread intestinal metaplasia [25]. By using linked color imaging (**f**) the gastric fundus [28], and (**g**) the antral mucosa appeared massively red [29]. (**h**) Reddish, depressed lesion observed in the greater curvature with conventional white light endoscopy 36 months after *H. pylori* eradication [23].

In the recent years, there was also an attempt to use more sophisticated tools to diagnose *H. pylori*. For example, Nakashima et al. developed an artificial intelligence approach, mimicking the brain neural network using BLI-bright and LCI. This method demonstrated to improve the accuracy and productivity of endoscopic examination, with respect to WLI, with a sensitivity for BLI-bright and for LCI of 96.7% [33]. In a recent meta-analysis, the artificial intelligence algorithm demonstrated to be an accurate tool for the prediction of *H. pylori* infection during endoscopic procedures, although, the authors concluded that the real application needs to be evaluated in clinical studies [34]. Figure 1 shows some gastric mucosa features associated with *H. pylori* infection in different studies.

### 2.2. Histology

The examination of gastric mucosal biopsy specimens remains the gold standard for the detection of *H. pylori*, with a sensitivity of 95% and a specificity of 98%. In addition, it enables the visualization of gastric morphology at any time. However, in order to obtain an accurate diagnosis, two antral biopsies, one from the gastric angulus, and two biopsies from the corpus, are necessary [35]. For example, in a series of 213 patients, detection of atrophic gastritis and intestinal metaplasia were missed in 8% and 3% of cases, respectively, when biopsy samples were not collected from the angulus [36]. As the widespread use of proton pump inhibitors (PPIs) may result in atypical presentation of gastritis, or in density variation of bacteria at different sites [37], the accuracy of histologic diagnosis of *H. pylori* infection can be improved by using special staining techniques, specific immune stain, or digital pathology [38,39]. However, a recent study reported a high percentage (94%) of *H. pylori* detection with the standard hematoxylin-eosin staining, compared with special staining [40].

### 2.3. Rapid Urease Test

Upper endoscopy also allows to collect biopsy specimens for urease testing. The method takes advantage from the presence of pre-formed urease by the organism and, in media containing urea, the enzyme releases ammonia, increasing the pH and resulting in a color change of the medium.

The urease test is rapid (RUT), easy to perform, highly specific, and inexpensive for *H. pylori* diagnosis. However, RUT requires a high density of bacteria, for example, in the standard commercial kits, at least a 10^4^ bacterial load in the gastric specimens is required [41]. False-negative results may occur with recent use of antibiotics, bismuth-containing compounds, PPIs, especially omeprazole and lansoprazole, and in children younger than five years [42]. To collect biopsies from the corpus, rather than from the antrum, or combining antral and corpus biopsies, has been shown to enhance RUT sensitivity [43,44]. On the other hand, Dechant et al. reported a higher sensitivity of RUT compared to histology for the detection of *H. pylori* infection in patients exposed to PPI or antibiotics [45]. Similar results were reported for a liquid RUT, the preOx-HUT, from a multicenter prospective study. Compared with histology, the preOx-HUT showed a sensitivity and specificity of 85% and 94%, respectively. More importantly, the concomitant PPI-use did not influence test accuracy [46]. In addition to false negative, false-positive RUT may also occur in the presence of urease positive bacteria [41]. The gastric samples used for RUT can be re-used for molecular testing in order to identify bacterial resistance. However, compared with histology, RUT does not allow to establish a correct follow up of the patient.

### 2.4. Culture

In addition to histological examination and RUT, upper endoscopy offers the opportunity to collect gastric specimens for bacterial culture, susceptibility testing and, eventually, organism genotyping. Although culture is highly specific, it has a low sensitivity, as *H. pylori* is difficult to grow, and experienced laboratories are required. Sensitivity may be improved by sending the specimen to the laboratory within 30 min from collection, using a pre-heated 35 °C blood agar (BD Diagnostics, Sparks, MD, USA) and a helicobacter-selective agar, containing the antibiotics colistin and polymyxin (Hy-Laboratories, Rehovot, Israel). A longer incubation period (14 days) in microaerophilic conditions (CampyGenTM, Oxoid, Hampshire, UK), at a temperature of 35 °C, the addition of hydrogen in the atmosphere, or to treat specimens with trypsin may be an additional shrewdness [47,48,49,50].

## 3. Non-Invasive Tests

Non-invasive tests can be divided into those able to detect an active infection, such as the urea breath test and stool antigen test, and those able to provide information on current or prior *H. pylori* infection, without discrimination.

### 3.1. Urea Breath Test

The ^13^C-urea breath test (UBT) is the non-invasive method of choice to determine *H. pylori* status when available. Similarly to RUT, the test takes advantage from the urease produced by the bacteria, which is able to hydrolyze urea generating CO_2_ and ammonia. The urea substrate is enriched with a labeled carbon isotope, that may be non-radioactive (^13^C) or radioactive (^14^C) and ingested, usually, with a test meal to prolong the permanence of urea in the stomach. Breath exhaled samples are collected in proper tubes before and after urea ingestion. Even though the dose of radiation is small in the ^14^C-UBT, the non-radioactive ^13^C test is routinely preferred. The test is also used to ascertain the eradication and it is recommended for the “test-and-treat” strategy in dyspeptic patients [1]. The test could also be successfully applied to patients with partial gastrectomy, especially when performed with the patient in the right position [51].

The ^13^C-UBT shows high sensitivity (95%) and specificity (95% to 100%) [52]. A review comparing diagnostic accuracy of non-invasive tests against histology, including 99 studies, reported a sensitivity of 94%, 92%, 84%, and 83% for the ^13^C-UBT, ^14^C-UBT, serology and for the stool antigen test, respectively, estimated at a specificity of 90% [53]. The high accuracy of ^13^C-UBT was also confirmed in a meta-analysis, including 15 studies performed in Asia. The sensitivity and specificity results were excellent, notwithstanding the heterogeneity observed across all studies [54].

The ^13^C-urea is available on the market in different formulations, such as powder, capsules and tablets ranging between 50 and 100 mg, however the cost may be expensive for low-income countries. Coelho et al. set up a cheaper Brazilian substrate for the UBT that showed a similar diagnostic accuracy compared with the commercial formulation, making the ^13^C-UBT potentially more widely available in the country [55]. To improve results, several test meals have been used in different studies across several countries, however, the citric acid or malic acid enhances ^13^C-UBT performance, increasing urease activity in the presence of bacteria [56]. For example, in dyspeptic patients chronically exposed to esomeprazole 40 mg daily, ^13^C-UBT with a test meal containing 5.5 g powder mixture of citric, malic and tartaric acid (Refex^®^), demonstrated to have a sensitivity and specificity of 92.45% and 97.96%, respectively, per-protocol analysis, after a one day break in medication [57]. However, quantitative results may be influenced by sex, age, body mass index; especially obesity, smoking, gastric atrophy and intestinal metaplasia, and even by socioeconomic status [58,59,60]. Although the most used cutoffs, expressed as delta over baseline (DOB), are 2‰, 2.4‰, 2.5‰ and 5‰ [61], in a large community-based intervention trial, conducted in Linqu County, China, on 21,639 subjects included in the analysis, the optimal DOB cutoff was reported to be 3.8‰ with 75 mg of ^13^C-urea [62]. Similarly, in a retrospective study performed on 234,831 patients, cluster analysis demonstrated that the ^13^C-UBT missed 2180 positive patients adopting the DOB cutoff of 3.5‰ recommended by the manufacturer [63]. Interestingly, an additional study reported that high DOB values may predict failure when a traditional triple therapy is used [64]. Recently, a study demonstrated that when shortening the testing time to 15 min by BREATHQUALITY UBT (AB Analitica, Padua, Italy), the resulting accuracy was comparable with 30 min standard testing time [65].

To analyze labeled ^13^CO_2,_ several detector devices are available on the market. One of the most recent, the BreathID Hp Lab System (Exalenz Bioscience Ltd., Modiin, Israel), was evaluated in a study in pre-treated patients and to assess *H. pylori* eradication; in both cases diagnostic accuracy was excellent, according to histology and RUT [66]. The performance of BreathID Hp was comparable with the IRIS-Doc2 (Wagner Analysen-Technik, Bremen, Germany, now Mayoly Spindler Group, Chatou, France) in a prospective study when the same test protocol was used [67]. Among the instruments suggested to provide ^13^C-UBT in-the-office, is the Otsuka device [68]. The instrument, through infrared spectroscopy, measures the ^13^CO_2_/^12^CO_2_ ratio directly from the bags used to collect breath samples. Although it is very easy to use, the UBiT kit; (Otsuka Pharmaceutical, Tokyo, Japan; cutoff value: 2.5‰) was criticized for its poor specificity in confirming *H. pylori* status after eradication [69]. The reliability of the UBiT kit was rescued by Ramirez-Lazaro et al. [70]. In their study, the authors demonstrated that false positive results obtained by UBiT were actually true positives, when the analysis was complemented by PCRs amplifying genomic ureA and 16S rDNA [70].

### 3.2. Stool Antigen Test

To culture *H. pylori* from feces is very difficult and time consuming [71], on the contrary non-invasive tests able to detect *H. pylori* antigen in stool specimens are simple to perform and large head-to-head comparisons with other tests demonstrated the high diagnostic accuracy of this approach [72]. The first to have been introduced was the Premier Platinum HpSA, Meridian Diagnostics, Inc., Cincinnati, OH, USA [73] and several assays are already available, the more recent ones are listed in Table 1.

Overall, stool monoclonal antibody tests are superior to polyclonal antibody tests and demonstrated a pooled sensitivity and specificity of 93% and 96%, respectively [79,80]. They also showed an excellent diagnostic accuracy in pediatric setting, especially when tests are ELISA based rather than immunochromatography based [81]. The use of the stool antigen test (or UBT) for the initial diagnosis of *H. pylori* infection and post-treatment (when endoscopy is not required), was recommended by a group of 11 experts at the Houston Consensus Conference [4].

The advantage of the UBT and of the stool antigen test is that they assess the overall content of the stomach, whereas the histology and RUT only assess a tiny biopsy specimen. Theoretically and practically, the UBT and stool antigen test are the best methods for detection of active *H. pylori* infection. However, any drug that diminishes *H. pylori* numbers below the threshold of detection can cause false negative results, particularly recent use of proton pump inhibitors, bismuth-containing compounds, or antibiotics.

### 3.3. Molecular Testing

**Figure 2:**
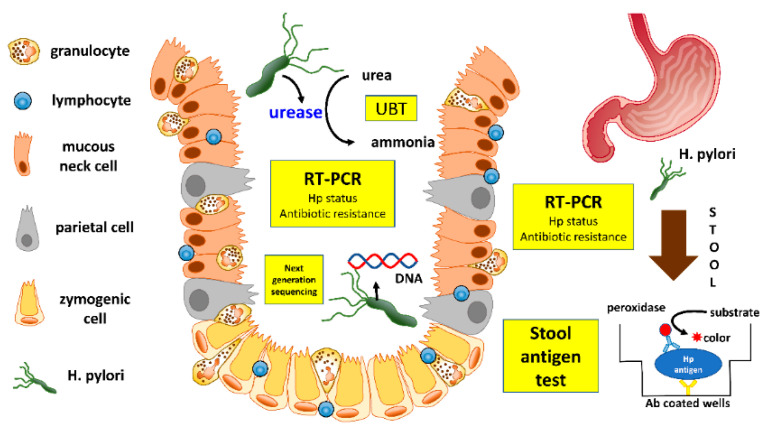
Invasive, non-invasive methods and molecular testing to detected *H. pylori* and its antibiotic resistance.

Molecular techniques should be preferred when available. The traditional or modified real-time (RT) PCR allows for the detection of bacteria, and to screen for antibiotic sensitivity [82,83,84]. Moreover, the real-time PCR proved to be more accurate when compared with other techniques for the detection of *H. pylori* in patients exposed to PPI [85], and was shown to be able to detect as few as 10 copies in adults [86] and children [87]. In addition to gastric biopsies, molecular tests can be applied to the gastric mucus present on biopsy forceps placed into water or into the RUT gel [88], and the dual-priming oligonucleotide-based multiplex PCR, performed on CLO^®^-test kits, proved to be superior to RUT and histology, in patients with a bleeding peptic ulcer [89]. Alternatively, molecular tests to detect *H. pylori* and its susceptibility to antibiotics can be performed on gastric juice [90,91,92]. A droplet-digital PCR may also be applied to formalin-fixed, paraffin-embedded gastric tissue to determine the presence of clarithromycin resistance [93], or by next generation sequencing to determine levofloxacin and tetracycline resistance [94] (Figure 2).

Several molecular tests have been developed in the last years to detect specific *H. pylori* antigens and/or resistance pattern in the stool (Table 2).

### 3.4. Serology

Unlike UBT and stool antigen testing, serology does not distinguish between an active or past infection, although in a recent study, antibody response to *H. pylori* proteins, such as VacA, GroEl, HcpC, CagA, Tip-α, HP1564, and HP0175 indicates an active *H. pylori* infection with a high diagnostic accuracy [102,103].

Detection of serum IgG against *H. pylori* is usually based on the enzyme-linked immunosorbent assays (ELISA). The latex immunoassay (Eiken Chemical Co, Ltd., or Denka Seiken Co, Ltd., Tokyo, Japan), is also employed with some advantage in terms of time consumed [104]. Several kits are available on the market and, overall, they are highly sensitive and specific. However, to maintain high diagnostic accuracy, serologic tests need to be validated locally [105], especially when the kit uses antigen strains from different geographic areas [106]. Because IgG titers decline slowly (over around six months), the test is not recommended to evaluate bacterial eradication after treatment. However, in the United States, despite the ACG and AGA guideline recommendations, serologic testing was the most commonly prescribed assay for the evaluation of *H. pylori* infection, until few years ago [107]. High serum antibody titers in subjects between 40 to 59 years old have been associated with the presence of gastric mucosa nodularity and or atrophy [108]. Similarly, serology positivity for CagA, Tip-α, HP0175, Omp, and HP0305 can predict an increased risk of atrophic gastritis or, more in general, precancerous lesions [103,109].

### 3.5. Tests on Plasma, Blood, Saliva and Urine

The GastroPanel^®^, especially the new-generation test, which assesses *H. pylori* antibodies and pepsinogen (PG) I plus PG II and gastrin-17 in the plasma simultaneously, is able to predict *H. pylori* infection and the presence of atrophic gastritis with a likelihood of 94–95% [110]. The test was reported by several authors as the most comprehensive non-invasive diagnostic test, as it avoids false and negative results, with respect to conventional tests [57,110,111,112,113]. For example, in a study performed in Korea, a decreased PG I/II ratio was significantly associated with chronic atrophic gastritis and intestinal metaplasia (*p* < 0.001) and, inversely, an increased ratio correlated with endoscopic findings, such as gastric and duodenal ulcer or nodular gastritis [111]. A similar association between a decreased PG I/II ratio and precancerous gastric conditions was also confirmed in several European countries, although the authors criticized the PG low specificity and its testing limitation for assessing gastric cancer risks [114]. Moreover, the measurement of PG I levels in the serum and the assessment of *H. pylori* infection may also be used to identify patients at high-risk for gastroduodenal injury induced by aspirin [115]. Interestingly, a cutoff value of PG I ≤ 31.2 ng/mL and PG I/II ratio ≤ 4.6 was able to discriminate for a past *H. pylori* infection in patients with Group A blood in a multicenter study [116].

A plasma sample also offers the opportunity to detect circulating microRNAs (miRNAs) by molecular techniques. For example, the expression of four miR-28-3p, miR-143-3p, miR-151a-3p and miR-148a-3p were shown to be associated with *H. pylori* infection [117].

In contrast, the accuracy of two plasma antibody test-systems (latex agglutination and ELISA) were suboptimal when compared with histology for gastric cancer screening [118].

IgG antibodies against *H. pylori* may also be detected in dried blood spots, saliva, and urine by ELISA, with good reported accuracy [119,120,121]. The diagnostic performance of a rapid urine test, based on immunochromatography (RAPIRUN by Otsuka Pharmaceutical Co., Ltd., Tokyo, Japan), was evaluated in a study conducted in Thailand. The accuracy of RAPIURIN was 89.4% compared with RUT (95.7%), histopathology (97.9%) and culture (97.9%), respectively [122]. In addition, a rapid urine IgG antibody test (u-HpELISA, Otsuka Pharmaceuticals Co., Ltd., Tokyo, Japan) was assessed in Japanese adolescents and the reported sensitivity and specificity were 85.7% and 100%, respectively [120]. Detection of *H. pylori* in the dental biofilm and in saliva samples, evaluated in dyspeptic children by RT-PCR targeting 16S rRNA and 23S rRNA genes, demonstrated to be superior to gastric biopsy specimens [123].

## 4. Summary

Physicians now have at their disposal a wide variety of diagnostic methods, which are classified into invasive and non-invasive. The choice of the test cannot prescind from the clinical scenario, local availability, knowledge of lab reliability and performance quality, costs, ongoing treatment, and patient desiderata. As a rule of thumb, it is important for the physician to confirm the diagnosis, to evaluate the presence of gastric lesions induced by the infection according to the patients’ clinical history, to offer *H. pylori* eradication therapy, and to check treatment success.

**Figure 3:**
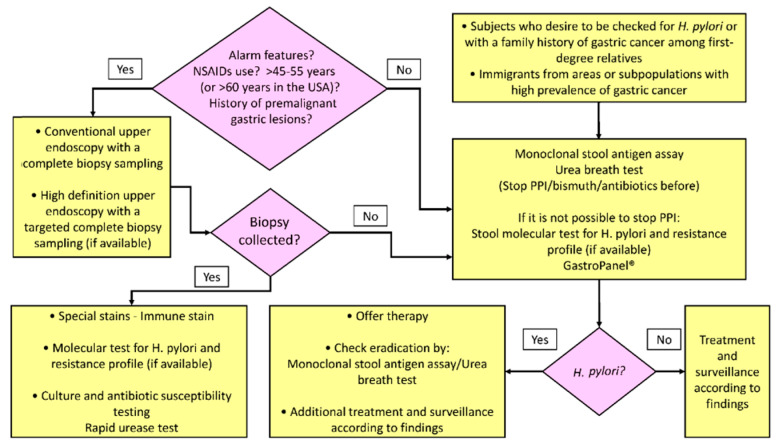
Diagnostic evaluation to detect *H. pylori* infection and its gastric mucosa sequelae.

Our approach to, and the extent of the diagnostic evaluation of a patient with uninvestigated dyspepsia is based, beyond the age of the patient on clinical presentation, on presence/absence of alarm features, ongoing treatment with NSAID and/or direct oral anticoagulants (Figure 3).

According to the recommendations of major gastroenterology societies, the age cut off may range from 60–65 years in patients from resource-rich countries with a low prevalence of gastric cancer, to 45–50 years or less in patients from countries or subpopulations with a high prevalence of gastric cancer [1,2,3,4]. Under these age thresholds, in the absence of alarm symptoms and use of NSAIDs or anticoagulants, we apply a “test and treat” strategy.

The best tests to non-invasively assess an active *H. pylori* infection are the ^13^C-UBT and the monoclonal stool antigen test, both of which are highly accurate. However, they may be falsely negative if the patient is under PPI or bismuth compound treatment or was exposed to antibiotic therapy within the month before testing. The molecular assay on stools should be preferred to evaluate *H. pylori* antibiotic resistance pattern when locally available.

If endoscopy is not required, the use of the stool antigen test or UBT is also recommended to ascertain eradication. For asymptomatic subjects who desire to be evaluated for *H. pylori* infection, we apply the same strategy.

In the case of a positive family history of gastric malignancy in successfully eradicated dyspeptic patients or asymptomatic subjects, after around six months we perform an upper endoscopy with multiple biopsies (at least 2 from the antrum, 2 from the angulus and 2 from the corpus), in order to assess the presence and the extent of precursor lesions of intestinal type gastric cancer, and to offer, accordingly, the best surveillance program [124].

For patients in whom an upper endoscopy is required, where available, advanced endoscopy techniques (for example NBI, BLI, LCI, etc.) should be preferred to conventional WLE endoscopy, especially for those patients/subjects with a high pretest probability to harbor premalignant lesions, such as those from countries or subpopulations with a high prevalence of gastric cancer, or individuals with strong familiarity for gastric malignancy, or those patients who need a strict endoscopy surveillance for previously diagnosed premalignant lesions.

High-definition endoscopy allows, in real time, the diagnosis of *H. pylori* infection, detection of premalignant and malignant gastric lesions and targeted mucosa biopsy sampling.

Gastric biopsy specimens obtained by high-definition or conventional endoscopy can be used for molecular testing to assess the presence of *H. pylori* and its antibiotic susceptibility profile in patients who are also under PPI treatment. This is particularly useful for those patients who cannot stop the PPI treatment (for instance because of on double antiplatelet treatments, or with a Zollinger Ellison syndrome or similar circumstances).

A RUT and culture would be a good option to detect *H. pylori* and to evaluate the antibiotic resistance profile, however they may result falsely negative in patients exposed to PPI/bismuth/antibiotic. Moreover, they do not allow for the evaluation of the gastric mucosa status.

Under specific conditions, despite the need of an upper endoscopy, it is mandatory to evaluate the risks and benefits to perform an invasive procedure to assess the presence of *H. pylori* infection and/or its sequalae. For instance, in very old or fragile patients, or in those with severe comorbidity, or healthy subjects from regions at low gastric cancer prevalence, but with gastric cancer familiarity, who refuse upper endoscopy, an evaluation with the GastroPanel^®^ may offer a comprehensive overview of the *H. pylori* and gastric mucosa status.

However, since the GastroPanel^®^ is rarely provided by the public health system, in this setting to test *H. pylori* status in the serum, plasma, saliva, blood or urine (based on what is locally available) may be an option. Evaluation of gastrin and PG I and PG II levels can supply a sort of home-made GastroPanel.

Serology testing does not have the ability to distinguish active from past infection. In addition, the positive predictive value of antibody testing is affected by the local prevalence of *H. pylori*, especially in those areas where the prevalence is inferior to 20%. Although some authors have suggested that quantitative serologic testing may be useful in documenting the infection clearance, this is not usually performed in clinical practice.

## Figures and Tables

**Table 1:** Most recent stool antigen tests and their reported sensitivity and specificity.

Brand	Based on	Sensitivity	Specificity	Reference
LIAISON *H. pylori* SA assay (DiaSorin, Saluggia, Italy)	chemiluminescent immunoassay	90.1 95.5	92.4 97.6	Ramirez-Lazaro et al., 2016 [70]
Genx *H. pylori* card test (Genx Bioresearch, Kocaeli, Turkey)	monoclonal immunochromatographic assay	51.6	96.0	Korkmaz et al., 2015 [74]
Uni-Gold™ *H. pylori* Antigen (Trinity Biotech, Bray, Ireland)	monoclonal lateral flow immunochromatographic assays	83.2	87–89.3	Lario et al., 2016 [75]
RAPID Hp StAR (Oxoid Ltd., Hampshire, UK)	monoclonal lateral flow immunochromatographic assays	94–95	77.1–84.7	Lario et al., 2016 [75]
ImmunoCard STAT! HpSA (Meridian Diagnostics, Cincinnati, OH, USA)	monoclonal lateral flow immunochromatographic assays	79–81.5	90.8–91.6	Lario et al., 2016 [75]
IDEIA HpStAR^®^; (ThermoFisher Sc., Waltham, MA, USA)	monoclonal antibodies and the ELISA technique	Before *Hp* treatment 93.6 After *Hp* treatment 100	Before *Hp* treatment 100 After *Hp* treatment 92.8	Moubri et al., 2018 [76]
Quick Chaser *H. pylori*^®^, QCP, Misuho Medy, Tosu, Japan)	immunochromatography	92.3		Kakiuchi et al., 2019 [77]
Vstrip^®^HpSA (Meridian)	immunochromatography	91%	97%	Fang et al., 2020 [78]
ImmunoCard STAT!^®^ Campy (Meridian)	immunochromatography	76.9%	97%	Fang et al., 2020 [78]

**Table 2:** Recent molecular assays available to detected *H. pylori* and its antibiotic resistance.

Molecular Test	*H. pylori* DNA Target	Reference
multiple genetic analysis system (MGAS)	16S rDNA and *ure*C	Zhou et al., 2015 [95]
allele-specific PCR	N87I mutation in the *gyr*A	Trespalacios et al., 2015 [96]
droplet-digital PCR (ddPCR)	*cag*A and its EPIYA phosphorylation motifs	Talarico et al., 2016 [84]
loop-mediated isothermal amplification (LAMP)	*ure*C gene	Yari et al., 2016 [97]
TaqMan RT-PCR	A2142C, A2142G and A2143G mutations	Beckman et al., 2017 [98]
droplet-digital PCR (ddPCR)	16S rDNA	Talarico et al., 2018 [93]
real-time PCR (THD fecal test^®^)	23S ribosomal RNA	Iannone et al., 2018 [99]
MagNA Pure 96 (Roche)	DNA	Clines et al., 2019 [100]
Amplidiag^®^ *H. pylori* + ClariR	*H. pylori* and CLA resistance mutations	Pichon et al., 2020 [101]
